# Tailored Gas Adsorption Properties of Electrospun Carbon Nanofibers for Gas Separation and Storage

**DOI:** 10.1002/cssc.202000520

**Published:** 2020-05-04

**Authors:** Ansgar Kretzschmar, Victor Selmert, Henning Weinrich, Hans Kungl, Hermann Tempel, Rüdiger‐A. Eichel

**Affiliations:** ^1^ Forschungszentrum Jülich GmbH Institute of Energy and Climate Research—Fundamental Electrochemistry (IEK-9) 52425 Jülich Germany; ^2^ RWTH Aachen University Institute of Physical Chemistry 52056 Aachen Germany

**Keywords:** adsorption, carbon, carbon dioxide capture, molecular sieves, nanofibers

## Abstract

Carbon nanofibers (CNFs) derived from electrospun polyacrylonitrile (PAN) were investigated with respect to their gas adsorption properties. By employing CO_2_ adsorption measurements, it is shown that the adsorption capacity and selectivity of the fibers can be tailored by means of the applied carbonization temperature. General pore properties of the CNFs were identified by Ar adsorption measurements, whereas CO_2_ adsorption measurements provided information about the ultramicroporosity, adsorption energies, and adsorption capacities. Ideal adsorbed solution theory (IAST) selectivities under practically relevant conditions were determined by evaluation of single‐component data for N_2_ and CO_2_. Especially for low carbonization temperatures, the CNFs exhibit very good low‐pressure adsorption performance and excellent CO_2_/N_2_ IAST selectivities of 350 at 20 mbar and 132 at 1 bar, which are attributed to a molecular‐sieve effect in very narrow slit pores. These IAST selectivities are some of the highest values for carbon materials reported in the literature so far and the highest IAST selectivities for as‐prepared, non‐post‐treated carbon ever.

## Graphical abstract

**Tailored adsorbents**: Carbon nanofibers (CNFs) are fabricated by carbonization of electrospun polyacrylonitrile fibers. Their gas adsorption properties, which are determined by the carbon interlayer distance, can be tailored by means of the carbonization temperature. Thus, CNFs with very good low‐pressure adsorption performance and excellent selectivity for CO_2_, which are attributed to a molecular‐sieve effect in narrow slit pores, can be obtained.

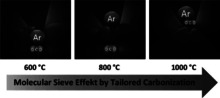

## Introduction

The scientific significance and public perception of anthropogenic climate change are increasing significantly,^1^ as the fraction of CO_2_ in the atmosphere has reached 407 ppm and is still growing. To attenuate the devastating effects of climate change, CO_2_ emissions must be reduced by any means. Two feasible ways to do so are to avoid the consumption of fossil fuels such as crude oil and natural gas, and to capture and store CO_2_ whenever possible. Although technologically and economically challenging, the latter may have benefits besides the reduction of CO_2_ emissions. Once separated from other gases, CO_2_ can chemically be transformed into value‐added organic compounds such as methanol^2^ and ethylene.^3^ For this, electrochemical CO_2_ reduction is particularly attractive due to its versatility^4^ and the possibility to combine both CO_2_ capture and utilization in one process. By transforming CO_2_ into synthetic fuels or raw organic chemicals, a closed carbon cycle leading to a CO_2_‐neutral economy can be established.

With the aim of efficient CO_2_ adsorption,^5^ a large number of different materials have been investigated, including metal–organic frameworks (MOFs),^6^, ^7^, ^8^ zeolites,^9^, ^10^, ^11^, ^12^ carbons,^13^, ^14^, ^15^, ^16^, ^17^ polymers,^18^ and functionalized silica.^19^ Among these materials, carbons offer several advantages over zeolites and MOFs, since they are widely abundant, cheap, easy to prepare, and comparatively insensitive towards contaminants such as water.^5^, ^20^, ^21^ Moreover, in contrast to polymers, carbons are mostly electrically conductive,^20^ which enables their application as current collectors in CO_2_ electrolyzers as well. However, two aspects that are lacking with respect to present carbon materials are decent adsorption capacity and CO_2_ selectivity at low pressure, which so far are significantly better for tailor‐made MOFs.^5^, ^21^

Owing to the numerous advantages, a large variety of carbons has been investigated in search of improved CO_2_ adsorption properties.^15^, ^16^, ^17^ Many of these were biomass‐based, for example, carbons that have been prepared from soy bean dreg,^22^ coconut,^23^ palm shell,^24^ bamboo,^25^ and many more. Biomass‐based carbons are fairly easy to prepare and cheap. However, tailoring their properties is demanding, since the chemical composition and structure (i.e., the pore system) are fixed by the precursor.^15^ In contrast, carbons may also be synthesized from polymers, which provide higher controllability of the chemical properties. This includes carbons obtained from melamine^26^ or urea‐based resins^27^ as well as carbonized polypyrrole,^28^ polyindole,^29^ poly(vinylidene fluoride),^30^ and polyacrylonitrile (PAN).^31^, ^32^, ^33^

With the aim of maximum CO_2_ adsorption capacity and selectivity, two main approaches are available for carbon materials. Firstly, nitrogen functional groups can be introduced into the carbon matrix,^34^ as a high nitrogen content is favorable owing to acid–base interactions between the adsorbent and CO_2_. The number and nature of nitrogen based functional groups are usually adjusted by means of nitrogen‐containing precursors or subsequent functionalization of the prepared carbons. However, the impact of certain nitrogen‐containing groups on the CO_2_ adsorption properties is still a matter of debate. Whereas Li et al. consider pyrrolic groups most important for CO_2_ adsorption,^35^ Kim et al. found a larger influence of pyridinic groups.^33^

Secondly, the physical surface area may be enlarged by processes such as KOH etching and CO_2_ activation to increase the number of bare interaction sites.^17^ Shen et al.^36^ used KOH to activate commercial PAN fibers, which had a BET area of 0.24 m^2^ g^−1^. By KOH activation, the BET area was enlarged to 2231.24 m^2^ g^−1^, which increased the CO_2_ capacity by a factor of ten to 4.5 mmol g^−1^.^36^

Besides the adsorption capacity, the selectivity is another important property, especially for practical applications. For instance, increasing the CO_2_/N_2_ selectivity of a typical material (CO_2_ adsorption capacity: 3 mmol g^−1^) from 50 to 100 can mitigate the cost for CO_2_ capture from flue gas by 20 %, that is, from 35 to 28 USD t^−1^.^37^

For high selectivity a narrow pore system rather than a high specific surface area is required to achieve a molecular‐sieve effect.^21^ Moreover, the selectivity can further be enhanced by the introduction of nitrogen functionalities, which may contribute to high selectivity due to selective interactions with CO_2_ as well.^21^

Indeed, PAN‐derived carbons have been under investigation for CO_2_ adsorption before and have mostly been obtained from bulk polymer,^38^, ^39^ wet spinning,^31^, ^35^, ^36^ or electrospinning.^33^ However, most of these carbons were post‐treated by various activation processes to achieve enhanced CO_2_ adsorption properties. Thus, surprisingly little information is available on CO_2_ adsorption on unmodified PAN‐derived carbon.

In this work, unmodified, electrospun, PAN‐derived carbon nanofibers (CNFs) were investigated as adsorbents for CO_2_, with the aim of deeper understanding of the adsorption processes on polymer‐based carbons. PAN‐derived CNFs are easy to prepare, even on a large scale, and contain a significant amount of nitrogen functionalities, if an appropriate carbonization temperature is applied.^40^ In electrospinning, a PAN polymer solution is spun in a controlled atmosphere under a high‐voltage electric field for the preparation of a carbon mat consisting of nonwoven fibers. Afterwards, the PAN polymer chains are stabilized and cross‐linked in air and carbonized in argon to yield carbon fibers in the submicrometer range, that is, CNFs, which have a very high surface area.^41^ The elemental composition and the pore properties of the as‐prepared CNFs are finely adjustable by means of the carbonization temperature, and thus excellent low‐pressure adsorption capacity and selectivity towards CO_2_ are attainable. Thus, the approach to prepare a highly selective carbon material proposed herein particularly abstains from excessive post‐treatment by keeping the synthesis procedure simple and, therefore, as cost‐efficient and scalable as possible. As a result, we report maximum CO_2_ adsorption capacities of 1.5 mmol g^−1^ at 100 mbar and 2.8 mmol g^−1^ at 1 bar. Moreover, by tailoring the ultramicropore system (micropores <0.7 nm)^42^ it is possible to achieve ideal adsorbed solution theory (IAST) selectivities of 350 at a low pressure of 20 mbar and 132 at 1 bar. Both values are very close to typical results for MOFs, among the highest values for carbon materials reported so far, and the highest values for unmodified carbons.

## Results and Discussion

### Fiber morphology, structure and chemistry

**Figure 1:**
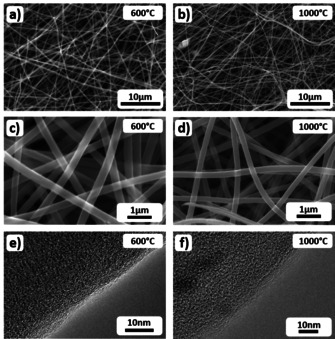
SEM (a–d) and TEM (e, f) images of electrospun, PAN‐derived CNFs. (a), (c), and (e) Fibers that were carbonized at 600 °C. (b), (d), (f) Fibers that were carbonized at 1000 °C.

By electrospinning and carbonization at different temperatures ranging from 600 to 1100 °C, CNF mats were prepared from a 10 wt % PAN solution (see Experimental Section). In the SEM images (Figure 1 a–d) the nonwoven fibers show an even surface and no preferred orientation at all carbonization temperatures. The fiber diameters are rather uniform and decrease only slightly from 250 to 220 nm in the investigated range of carbonization temperatures. Moreover, the TEM images in Figure 1 e and f reveal a slightly ordered carbon structure, which shows increased surface roughness for higher carbonization temperatures. A detailed in situ TEM analysis under vacuum of the effect of the carbonization temperature on the CNFs has been published elsewhere.^43^

To analyze the chemical composition of the CNFs, CHNO elemental analysis was performed (Table 1). The carbon content of the CNFs increases continuously for increasing carbonization temperature from 600 to 1100 °C with a larger step from 77.6 wt % (900 °C) to 91.5 wt % (1000 °C). Simultaneously, the nitrogen content of the carbonized fibers decreases almost linearly from 23 to 7 wt %. This correlation fairly matches the expectations based on the literature, in which 17 wt % nitrogen for 700 °C^44^ and 5.8 wt % nitrogen for 1000 °C^45^ were reported for carbonized PAN. In contrast to carbon and nitrogen, the oxygen content remains almost constant between 10 and 12 wt % in the temperature range from 600 to 900 °C, which indicates higher thermal stability of the remaining oxygen‐containing functional groups. Above 900 °C the oxygen content decreases to 0.8 wt % for a carbonization temperature of 1100 °C. It is expected that the majority of the oxygen‐containing groups in the cross‐linked fibers already decomposed and released oxygen as water at lower temperatures of 300–400 °C.^45^ Furthermore, the decrease of the hydrogen content from 2.7 to 0.4 wt % indicated progress of the carbonization. In fact, at higher temperatures C−H bonds are thermally destroyed and cross‐linking processes between carbon atoms take place.^46^

**Table 1:** Elemental composition of CNFs determined by CHNO analysis.

Carbonization temp.	Elemental composition [wt %]			
[°C]	C	N	O	H
250	52.2	22.6	24.1	2.7
600	63.5	23.0	11.8	2.1
700	67.9	19.9	11.1	1.8
800	72.6	16.2	10.6	1.4
900	77.6	11.6	9.5	1.2
1000	91.5	7.1	3.1	0.4
1100	95.8	3.7	0.8	0.5

In addition to the elemental analysis, X‐ray photoelectron spectroscopy (XPS) was performed to characterize the surface composition and the functional groups of the individual fiber mats (for spectra and integration, see Figures S2 and S3 in the Supporting Information). Moreover, XPS also yields data regarding the elemental composition (Table 2). On comparing the data from the CHNO method and XPS, it can generally be observed that the individual results show similar trends regarding the carbon and nitrogen contents. Although comparing the exact numbers is not possible, as XPS data is given in atom % and CHNO data in wt %, the difference is small for light elements with similar atomic mass. However, the XPS data show higher absolute values for carbon (76.5–95.1 atom %) and lower absolute values for nitrogen (19.7–3.1 atom %) and oxygen (3.8–1.8 atom %). In addition, the sudden jump in carbon content between the materials carbonized at 900 and 1000 °C is less pronounced for the XPS results as compared with the findings obtained by the CHNO method. In contrast to the results provided by combustion elemental analysis (CHNO), the surface‐oxygen fraction determined by XPS is far lower and does not exceed 4 atom %. Furthermore, the oxygen content does not show any trend depending on the carbonization temperature. This suggests that most of the oxygen that was detected by the CHNO method is trapped inside the fibers and is not accessible to XPS, as this technique is surface‐sensitive, limited to a penetration depth of a few nanometers,^47^ and also depends on the angle of incidence.^48^ However, the difference between elemental analysis and XPS results can also be explained by the experimental procedure, as the sample may have contained adsorbed CO_2_ or O_2_ during the elemental analysis, whereas XPS is performed under ultrahigh vacuum. Furthermore, the indirect measurement procedure for the determination of oxygen in the elemental analysis (see Experimental Section) could cause deviations.

**Table 2:** Elemental composition of CNFs determined by XPS analysis.^[a]^

Carbonization temp.	Elemental composition [atom %]		
[°C]	C	N	O
600	76.5	19.7	3.8
700	80.4	16.8	2.8
800	84.1	13.7	2.2
900	86.6	11.0	2.4
1000	92.6	4.3	3.1
1100	95.1	3.1	1.8

[a] Relative error=±15 %.

**Figure 2:**
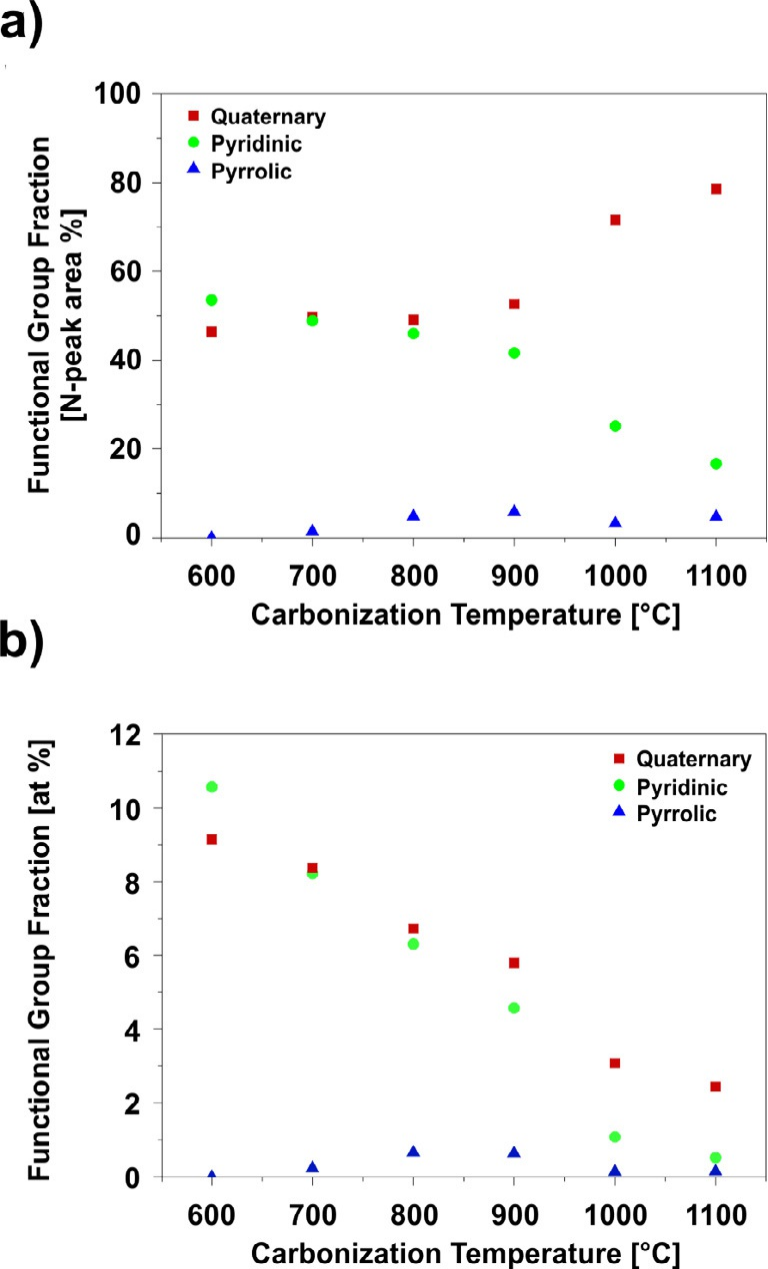
XPS analysis of electrospun, PAN‐derived CNFs carbonized at 600–1100 °C (for spectra and integration, see Figures S2 and S3). (a) Functional surface group fraction normalized to nitrogen peak area. (b) Total fraction of surface atoms.

Besides the elemental composition, XPS measurements also provide information on the functional groups on the material surface. Especially nitrogen‐containing functional groups are of interest for CO_2_ adsorption owing to their basic nature^20^, ^27^, ^49^ and their positive influence on weak hydrogen‐bonding interactions between CO_2_ and hydrogen on the carbon surface.^22^ Accordingly, the fractions of different nitrogen species were evaluated in dependence on the applied carbonization temperature. Figure 2 a depicts the XPS data normalized to the nitrogen peak area, and Figure 2 b shows the same data multiplied by the total nitrogen content from Table 2, which provide the overall contribution of the nitrogen‐containing functional groups to the atomic surface composition. In Figure 2 a, quaternary and pyridinic nitrogen are the dominant species. In contrast, pyrrolic nitrogen as the third potentially important species is almost absent. For a comparatively low carbonization temperature of 600 °C, the numbers of quaternary and pyridinic groups are almost equal, with a small advantage for the latter. However, with increasing carbonization temperature, the ratio of quaternary nitrogen to pyridinic nitrogen increases, with a ratio of approximately 4:1 at 1100 °C. This can be explained by the fact that pyridinic nitrogen in carbonized PAN is transformed into quaternary nitrogen at high temperatures.^50^ At the same time, the fraction of pyrrolic nitrogen increases for increasing carbonization temperatures from 600 to 800 °C and, besides a small variation, remains constant and always less than 5 % for carbonization temperatures above 800 °C.

Looking at the fraction of nitrogen moieties in the total number of surface atoms in Figure 2 b reveals that, even though quaternary nitrogen becomes more dominant, the absolute number of both quaternary and pyridinic nitrogen species continuously decreases from about 10 atom % to less than 2 atom % with increasing carbonization temperature. A possible reason is the decomposition of functional groups to N_2_, HCN, and NH_3_.^50^, ^51^ Furthermore, the number of pyrrolic nitrogen moieties shows a maximum at carbonization temperatures of 800 and 900 °C, but their fraction relative to the total number of surface atoms remains below 1 atom % and therefore most probably does not play a significant role in CO_2_ adsorption.

### Ar adsorption, surface area, and pore structure

**Figure 3:**
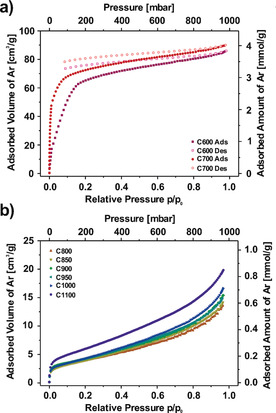
Ar adsorption isotherms of electrospun, PAN‐derived CNFs at 87 K. (a) Materials carbonized at 600 and 700 °C (C600 and C700). (b) Materials carbonized at 800–1100 °C (C800–C1100). In (b) the desorption branches are not shown for the sake of clarity, but can be found in Figure S4.

Besides functional groups, the pore system of a material is considered to be an important factor for CO_2_ adsorption,^49^ since a larger surface area increases the number of potential adsorption sites. Furthermore, narrow pores are highly beneficial to low‐pressure adsorption, as the adsorption potentials of opposing pore walls overlap.^42^, ^52^ To investigate the pore structure of the CNFs, static Ar adsorption measurements were performed at 87 K. The resulting isotherms are shown in Figure 3. For clarity, the results for the materials carbonized at 600 and 700, and 800–1100 °C are shown separately in Figure 3 a and b, and can be explained as follows. On carbonizing at 600 and 700 °C, the materials exhibit type I isotherms,^42^ which are typical of microporous adsorbents. However, the two isotherms are not in equilibrium, as the desorption branches do not meet the adsorption branch again at low relative pressures, even though the isotherms were measured with the highest technically possible equilibration parameters. This pseudo‐irreversibility of the Ar adsorption implies kinetic hindrance, which is known to occur when ultramicropores smaller than 0.45 nm are present.^53^ The isotherms both show a similar total adsorbed amount of Ar of 4 mmol g^−1^ at ambient pressure (1000 mbar). The only significant difference between the two materials is the slope of the isotherm at relative pressures below 0.1. Here, the much steeper slope of the material carbonized at 700 °C indicates narrower micropores or a higher adsorption energy.

In contrast to the under‐equilibrated isotherms of the materials carbonized at 600 and 700 °C, those carbonized at 800 °C and above exhibit a well‐equilibrated type II isotherm without any hysteresis (Figure 3 b), which is expected for nonporous materials or materials that do not have pores accessible to Ar. Furthermore, whereas the shape remains very similar for all materials, the overall adsorbed amount of Ar at 1000 mbar slightly increases from 0.6 to 0.9 mmol g^−1^, which is most probably due to slightly decreasing fiber diameter with increasing carbonization temperature.

From the Ar adsorption isotherms, BET areas were calculated (Table 3). The materials carbonized at 600 and 700 °C have BET areas of approximately 250 m^2^ g^−1^, which is comparatively high, as they were not activated by any additional reactant. Nevertheless, the value is low compared to those of chemically activated carbons, which easily exceed values of 1500 m^2^ g^−1^.^31^, ^36^, ^38^, ^39^ However, both results should only be considered as rough estimates, since the recorded isotherms were not fully equilibrated. In contrast, the materials carbonized at 800–1100 °C have surface areas of approximately 15 m^2^ g^−1^, which is close to the expected value of 9 m^2^ g^−1^ for nonporous fibers with a diameter of 200 nm. Overall, the surface areas show a slight trend to higher values for increasing carbonization temperatures, as observed for the adsorbed gas volumes at ambient pressure.

**Table 3:** BET data of CNFs.

Carbonization temp. [°C]	BET surface area [m^2^ g^−1^]
600	259^[a]^
700	249^[a]^
800	13.4
825	12.4
850	13.1
875	13.2
900	14.6
925	15.0
950	14.3
975	16.0
1000	14.8
1100	20.0

[a] Under‐equilibrated isotherm.

### CO_2_ adsorption and micropore structure

**Figure 4:**
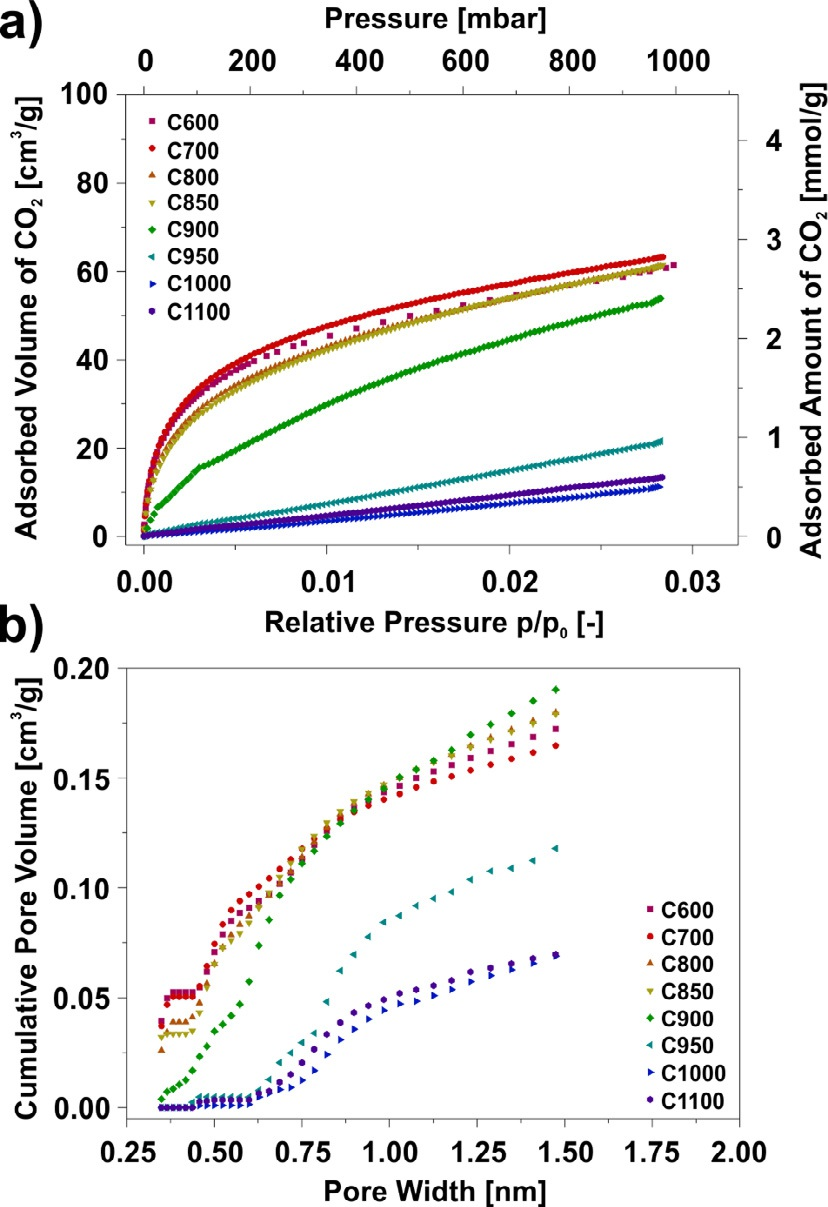
CO_2_ adsorption properties at 273 K of electrospun, PAN‐ derived CNFs carbonized at 600–1100 °C (C600–C1100). (a) CO_2_ adsorption isotherms (desorption branches not shown for clarity, see Figure S6). (b) Cumulative pore size distributions obtained by MC calculations.

CO_2_ adsorption measurements were performed to study potential ultramicroporosity and general CO_2_ adsorption properties of the CNFs. The CO_2_ adsorption isotherms shown in Figure 4 a were obtained at 273 K. (For the sake of clarity, desorption branches and the results for the materials carbonized at 825, 875, 925, and 975 °C are not shown here, but can be found in Figure S6). Figure 4 b shows the cumulative pore size distributions, which were obtained from the isotherms shown in Figure 4 a.

In Figure 4 a the individual CO_2_ adsorption isotherms show marked changes in shape depending on the applied carbonization temperature. The materials carbonized at 600–850 °C show a steep increase of the adsorbed amount of CO_2_ at low pressures, leading to 0.85 mmol g^−1^ and 1.3 mmol g^−1^ at pressures as low as 25 and 75 mbar, respectively, for 600 °C, which is a remarkable, practically relevant result for carbon. In fact, excellent low‐pressure performance is a key requirement for gas separation applications, for example, CO_2_ separation from flue gas with a low relative pressure of 8–13 % for CO_2_. (Note: unlike the Ar measurements, the materials carbonized at 600 °C and 700 °C were evaluated in equilibration due to the higher measurement temperature used for CO_2_ adsorption.^54^) At higher pressures, the isotherm slope for the material carbonized at 600 °C decreases and results in a final amount of 2.7 mmol g^−1^ at 1 bar. In contrast, the material carbonized at 900 °C shows a smaller initial slope of the CO_2_ adsorption isotherm, but only a slightly reduced amount of adsorbed CO_2_ of 2.4 mmol g^−1^ at 1 bar. At higher carbonization temperatures of 950–1100 °C the isotherms tend to become almost linear and the total adsorbed amount of CO_2_ at 1 bar remains below 1.0 mmol g^−1^. Furthermore, the adsorption kinetics for materials that were carbonized between 950 and 1100 °C appear to become slower, since the difference between adsorption and desorption branch increases with increasing carbonization temperature (see Figure S6).

For an improved overview of the individual adsorption properties, the adsorbed amounts of CO_2_ at certain pressures are given in Table S2 in comparison with those of some commercial carbons. At a pressure of 50 mbar, the adsorbed amount of CO_2_ for the material carbonized at 600 °C is more than 50 times higher than that of the material carbonized at 1000 °C. However, at ambient pressure, the amount of adsorbed CO_2_ is only about five times higher. Nevertheless, in contrast to the commercial carbons chosen for comparison, materials carbonized at temperatures between 600 and 875 °C show excellent low‐pressure adsorption performance, regardless of their comparatively low BET area. Black Pearls 2000, for example, has a BET surface area greater than 1500 m^2^ g^−1^ and adsorbs 4.3 mmol g^−1^ CO_2_ at ambient pressure, but its CO_2_ uptake at 50 mbar is only about half of those of the materials carbonized at 600–875 °C. Furthermore, Super P nonporous carbon and graphene platelets with a medium scaled BET area of 250 m^2^ g^−1^ also do not reach the adsorbed amounts of the investigated CNFs, neither at low nor at ambient pressure. Thus, the electrospun CNFs offer superior adsorption properties for CO_2_ at low pressures in comparison with other carbons (see also Table S1), which favor application in separating CO_2_ from gas mixtures with low CO_2_ concentrations.

To elucidate the origin of the superior adsorption properties, pore size distributions were derived from the individual CO_2_ isotherms by standard Monte Carlo (MC) calculations. The cumulative pore size distributions are shown in Figure 4 b. The materials carbonized at 600–850 °C appear to exhibit a significant amount of pores that are smaller than the calculation limit of 0.35 nm. This is indicated by the fact that the cumulative pore volume curves do not start at 0 cm^3^ g^−1^, but slightly above at 0.05 cm^3^ g^−1^.

Overall, the pore volume of ultramicropores smaller than 0.40 nm decreases with increasing carbonization temperature. Moreover, larger ultramicropores are present as well, reaching a total volume of 0.1 cm^3^ g^−1^ for the materials carbonized at 600–850 °C. In contrast, at a carbonization temperature higher than 950 °C the materials no longer have a significant measurable specific ultramicropore volume, but still show some supermicroporosity (0.7–2.0 nm).^42^

The slit‐pore width of 0.35 nm that was observed for the materials carbonized at 600–850 °C, is very close to the interlayer distance in graphitic (0.3354 nm) or turbostratic carbon (0.34 nm),^55^ which implies that the observed porosity in this range can be attributed to interlayer spaces of the carbon. In addition, the observed width range of the ultramicropores is not only close to the interlayer distance of graphite, but also matches the kinetic diameters of technically relevant gases. Indeed, CO_2_ has a kinetic diameter of 0.330 nm,^56^ whereas N_2_ and Ar are slightly larger (0.364^56^ and 0.340 nm,^54^ respectively). Therefore, a molecular‐sieve effect appears to be a reasonable explanation for the excellent CO_2_ adsorption capability compared with Ar.

An overview of the pore volumes and other textural properties such as the micropore surface area *S*_micro_ obtained by MC and Dubinin–Radushkevich (DR) calculations is given in Table 4 in comparison with those of commercial carbon materials. For carbonization temperatures of 600–900 °C the CNFs exhibit micropore surface areas of approximately 600 m^2^ g^−1^ (MC), which decrease to about 150 m^2^ g^−1^ for a carbonization temperature of 1000 °C and above. The DR results support the MC values. However, the DR values are about 25 % lower, which is suggested to be due to limitations of the DR equation regarding the heterogeneity of surface chemistry or texture.^57^ Furthermore, it is notable that even materials with a very low BET area of 15 m^2^ g^−1^ show a significant micropore surface area of more than 600 m^2^ g^−1^ (e.g., at 800 °C), which is, again, a hint that the ultramicroporosity of these materials is not accessible to Ar. Similar to the trend of the micropore surface area, the overall pore volume (MC) decreases from 0.190 cm^3^ g^−1^ (900 °C) to 0.07 cm^3^ g^−1^ (1000 °C), which is far less significant than the decrease in the adsorbed amount of CO_2_ at low pressures mentioned above. However, this trend complies better with the overall adsorbed amounts of CO_2_ at 1 bar, which was found to be five times higher for a carbonization temperature of 600 °C than for 1000 °C. This observation can be explained by the fact that larger pores are only filled at higher pressures. The DR micropore volumes are comparable to those obtained by MC calculations. Interestingly, the decrease in pore volume at 900 °C is sharper (0.195 cm^3^ g^−1^ at 875 °C to 0.06 cm^3^ g^−1^ at 925 °C) and slightly shifted towards lower carbonization temperatures when determined by DR, although both data sets were derived from the same raw data.

**Table 4:** Textural properties (micropore surface area *S*_micro_, cumulative pore volume at different thresholds) and adsorption energy derived from CO_2_ adsorption experiments on electrospun, PAN‐derived CNFs by Monte‐Carlo (MC) simulations and Dubinin–Radushkevich (DR) calculations.

Carbonization temp.	*S*_micro_ (MC)	*V*_<0.4 nm_ (MC)	*V*_>0.4 nm_ (MC)	*V*_tot_ (MC)	*S*_micro_ (DR)	*V*_tot_ (DR)	*E*_ads_ (DR)
[°C]	[m^2^ g^−1^]	[cm^3^ g^−1^]	[cm^3^ g^−1^]	[cm^3^ g^−1^]	[m^2^ g^−1^]	[cm^3^ g^−1^]	[kJ mol^−1^]
600	626	0.053	0.119	0.172	453	0.170	36.0
700	618	0.051	0.114	0.165	484	0.182	34.9
800	615	0.039	0.141	0.180	465	0.175	32.8
825	599	0.039	0.133	0.172	460	0.173	32.9
850	610	0.034	0.145	0.179	468	0.176	32.2
875	656	0.035	0.156	0.191	519	0.195	31.9
900	551	0.011	0.179	0.190	422	0.158	26.6
925	318	0.004	0.132	0.136	160	0.060	23.2
950	274	0	0.118	0.118	146	0.055	22.5
975	160	0	0.071	0.071	90	0.034	21.7
1000	151	0	0.069	0.069	95	0.036	20.3
1100	165	0	0.070	0.070	104	0.039	21.9
							
Reference samples:^[a]^							
Super P^[b]^	299	0	0.134	0.134	212	0.080	19.4
graphene platelets^[c]^	321	0.004	0.126	0.130	244	0.092	23.8
Black Pearls 2000^[d]^	1173	0.007	0.470	0.477	936	0.351	23.0

[a] The following samples are included for reference purposes. [b] Imerys. [c] Aldrich, 300 m^2^ g^−1^. [d] Cabot.

Black Pearls 2000 has a micropore surface area and a micropore volume that are twice as high as those of the material carbonized at 600 °C (Table 4). The latter is in very good agreement with the doubled CO_2_ loading at ambient pressure. On the other hand, Super P and graphene platelets have lower values, which correspond to their CO_2_ adsorption capacity at 1 bar as well.

Besides micropore surface area and micropore volume, the DR method allows one to obtain adsorption energies for CO_2_, which are also listed in Table 4. For a carbonization temperature of 600 °C, the adsorption energy is 36.0 kJ mol^−1^ and constantly decreases with increasing carbonization temperature to 31.9 kJ mol^−1^ for the material carbonized at 875 °C. Above 875 °C, the adsorption energy decreases to 23.2 kJ mol^−1^ for 925 °C and remains fairly constant at approximately 20 kJ mol^−1^ for higher carbonization temperatures. For further investigation, the isosteric heats of adsorption were calculated as well. The heats of adsorption are shown in dependence on the carbonization temperature and CO_2_ loading in Figure S8. For the materials carbonized at 600–800 °C the isosteric heats of adsorption are approximately 40 kJ mol^−1^ for small amounts of CO_2_ and 25 kJ mol^−1^ for high CO_2_ loadings. For carbonization temperatures of 1000 and 1100 °C the isosteric heat decreases to 10 and 5 kJ mol^−1^, respectively. An increase in adsorption energy can be caused either by a stronger chemical interaction between functional groups or by an overlap of pore‐wall potentials in narrow pores. However, since the number of functional groups and the number of ultramicropores increase with decreasing carbonization temperature, clear separation of the two effects is not possible.

**Figure 5:**
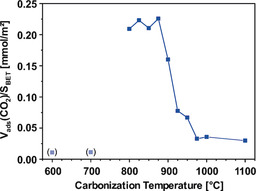
Ratio of adsorbed CO_2_ to BET area (Ar) for electrospun, PAN‐derived CNFs in dependence on the carbonization temperature. Data points in parentheses were obtained from underequilibrated isotherms.

By relating Ar and CO_2_ adsorption measurements with each other, it is possible to calculate a surface affinity towards CO_2_, that is, by dividing the adsorbed amount of CO_2_ by the BET surface area. On evaluating the surface affinities towards CO_2_, it can clearly be observed in Figure 5 that CO_2_ adsorption is highly favored in a carbonization temperature range of 800 °C to 900 °C. For these materials, the calculated surface affinities reach extremely high values of 0.2 mmol m^−2^ and drop by one order of magnitude for a carbonization temperature of 975 °C and above, due to the significantly lower CO_2_ adsorption capacity. For the materials carbonized at 600–700 °C the surface affinities are lower than 0.03 mmol m^−2^, due to the larger accessible surface areas, which were, however, obtained from under‐equilibrated isotherms.

Comparing the results in Figure 5 with literature data reveals that the investigated CNFs have remarkable properties. In fact, most carbons suggested for CO_2_ adsorption show significantly lower surface affinities, typically less than 0.005 mmol m^−2^, due to the very high BET areas of the corresponding materials (Table S1). Taking the very narrow pore widths into account, the extraordinary surface affinity implies that the superior adsorption properties at low pressures and the very high selectivity towards CO_2_ can be attributed to a molecular‐sieve effect. For carbonization temperatures of 600 and 700 °C, the narrowest micropores are accessible to both Ar atoms and CO_2_ molecules. On carbonizing between 800 and 875 °C, larger Ar atoms can no longer penetrate the shrinking pores, whereas CO_2_ adsorption is still possible. Furthermore, on further increasing the carbonization temperature, the micropores become too narrow to allow either CO_2_ or Ar adsorption, and the adsorption capacity decreases drastically. To substantiate the hypothesis that a molecular‐sieve effect is a major driver of the observed adsorption behavior of Ar and CO_2_, additional measurements on commercial zeolite molecular sieves with defined pore structure were conducted. These measurements along with a short interpretation are shown in Figure S7.

### Microporosity versus surface functionality

**Figure 6:**
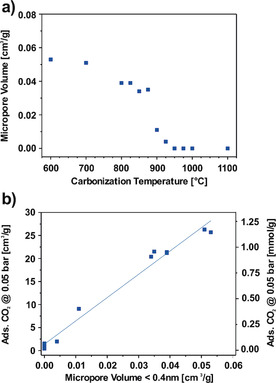
CO_2_ adsorption properties at 273 K of electrospun, PAN‐derived CNFs carbonized at 600–1100 °C. (a) Effect of the carbonization temperature on the available micropore volume. (b) Relation between the micropore volume (<0.4 nm) and the CO_2_ adsorption properties at 0.05 bar.

Besides microporosity, surface functionality is a second main factor influencing the CO_2_ adsorption capacity of carbon materials^34^ that can explain an increase in adsorption energy and surface affinity. Therefore, it is discussed in competition with the effect of pore shrinkage below. To this end, Figure 6 a shows the micropore volume for pores narrower than 0.4 nm for all materials given in Table 4. Figure 6 a reveals that the ultramicropore volume behaves similarly to the carbonization‐temperature‐dependent surface affinity shown in Figure 5. For materials carbonized at 600 and 700 °C, the volume of ultramicropores smaller than 0.4 nm is 0.05 cm^3^ g^−1^, whereas materials carbonized at 800–875 °C exhibit 0.04 cm^3^ g^−1^. Then, in a carbonization temperature range of only 100 °C, the micropore volume drops to 0 cm^3^ g^−1^ for a carbonization temperature of 950 °C and beyond.

Furthermore, Figure 6 b correlates the adsorbed amount of CO_2_ at 50 mbar with the volume of micropores smaller than 0.4 nm. Thus, it can be concluded that the volume of ultramicropores smaller than 0.4 nm is a major driver for the adsorbed amount of CO_2_ at 50 mbar. Indeed, whereas a material with an ultramicropore (<0.4 nm) volume of 0 cm^3^ g^−1^ adsorbs almost no CO_2_, a material with an ultramicropore (<0.4 nm) volume of 0.05 cm^3^ g^−1^ already leads to 1.1 mmol g^−1^ of adsorbed CO_2_ at 50 mbar. The correlation between ultramicropore (<0.4 nm) volume and adsorbed amount of CO_2_ is almost linear. This observation is a strong hint that, at the given pressure of 50 mbar, the ultramicropores are mainly responsible for the adsorption of CO_2_, rather than the open surface, which is in accordance with results previously described in the literature.^58^, ^59^, ^60^, ^61^

**Figure 7:**
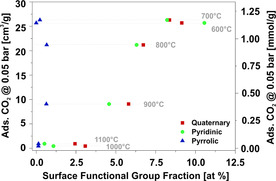
Influence of the nitrogen functional groups on the CO_2_ adsorption capacity on electrospun, PAN derived carbon nanofibers at 0.05 bar.

To study the correlation between nitrogen functional groups and the CO_2_ uptake, the adsorbed amount of CO_2_ at 50 mbar is plotted as a function of the fraction of surface nitrogen functional groups determined by XPS in Figure 7. From Figure 7 it can be deduced that there is a restrained correlation between pyridinic and quaternary groups and the adsorbed amount of CO_2_. It appears that in the range of carbonization temperatures between 700 and 1000 °C, both groups facilitate CO_2_ adsorption. This is confirmed by the correlation with the nitrogen content determined by elemental analysis (Table 1), since the adsorbed amount of CO_2_ is higher for the materials with a higher nitrogen content. Nevertheless, the correlation between adsorbed amount of CO_2_ at 50 mbar and the nitrogen functional groups is less clear than that between the adsorbed amount of CO_2_ and the ultramicropore volume. In contrast to pyridinic and quaternary moieties, the amount of pyrrolic nitrogen is too small to show a significant effect on CO_2_ adsorption, and no clear trend is visible. Similar results for the relation between CO_2_ adsorption and nitrogen content have been reported by Zhang et al. for polyaniline‐based carbons.^58^ They described a positive influence of nitrogen on CO_2_ adsorption, albeit with significant scattering of data points.

In general, it is difficult to distinguish between the effect of microporosity and nitrogen surface groups on the CO_2_ adsorption performance. Both parameters change continuously over the studied range of carbonization temperatures; thus, the influence of both might overlap and result in misleading correlations, although previous studies found a synergetic effect of microporosity and N doping.^62^ In addition, the influences of microporosity and functional groups cannot be discussed independently, since it is not possible to decrease the nitrogen content without influencing the microporosity for the same polymer and same preparation procedure, respectively. As a reason, we assume that nitrogen atoms act as structure‐disturbing heteroatoms, the decreasing number of which with increasing carbonization temperature results in less disturbance and, therefore, shrinkage of the carbon interlayer distance (i.e., narrower micropores).

**Figure 8:**
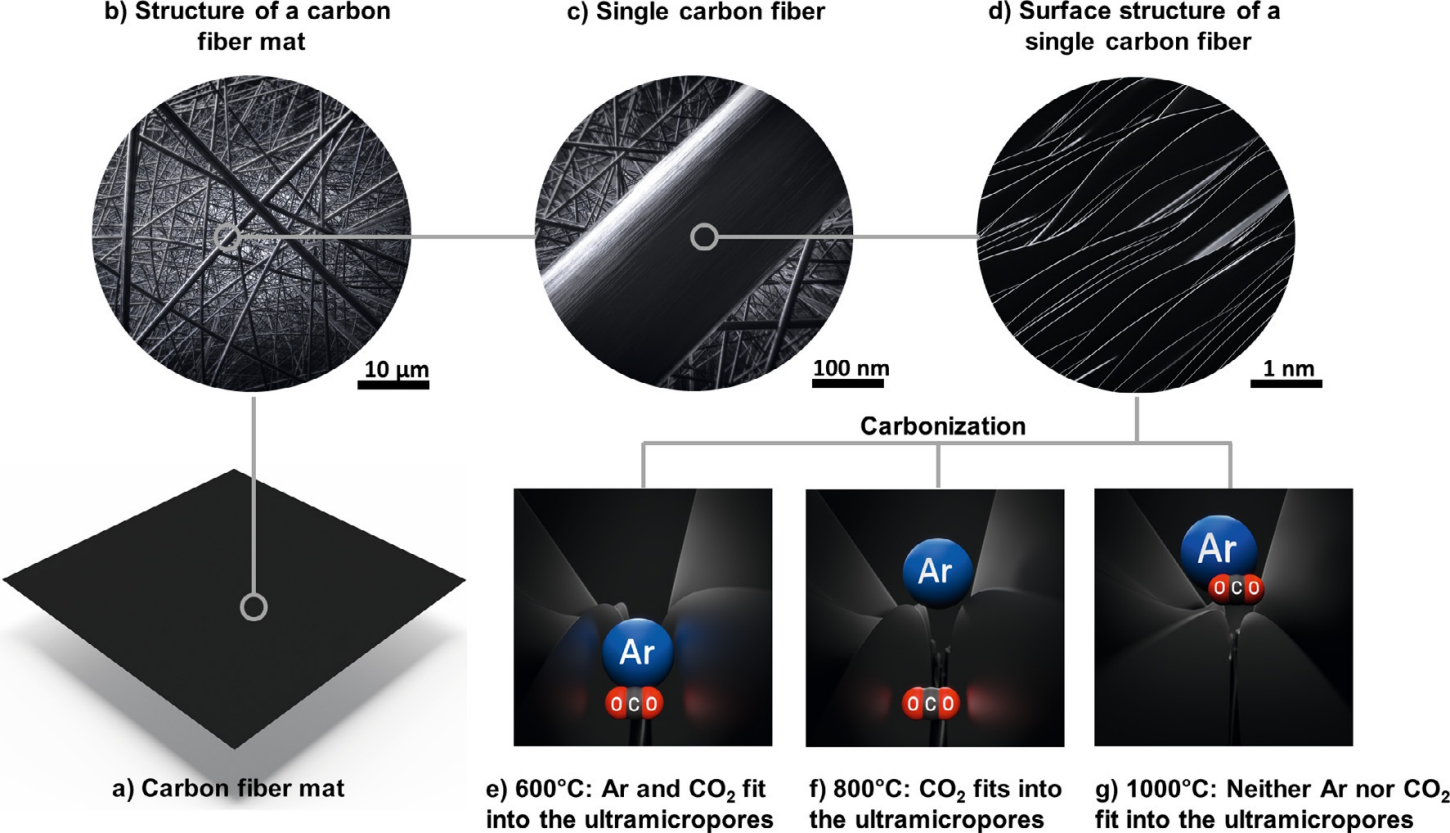
Overview of the Ar and CO_2_ adsorption properties of CNFs carbonized at 600, 800, and 1000 °C. Schematic of Ar and CO_2_ adsorption on electrospun, PAN‐derived carbon nanofibers carbonized at various temperatures ranging from 600 to 1100 °C. (a) Schematic of an as‐prepared, electrospun carbon fiber mat. (b) Zoom of the carbon fiber mat showing randomly oriented carbon fibers. (c) Zoom of the carbon fiber mat showing a single, as‐prepared carbon fiber with homogeneous and smooth surface. (d) Detailed view of a single carbon fiber, showing individual carbon layers, which act as slit pores with a varying slit pore width tailored by the applied carbonization temperature. (e–g) Schematic explanation of the excellent CO_2_ adsorption on the investigated CNFs. Owing to the decreasing carbon interlayer distance, the adsorption of individual gas molecules is prevented by size exclusion, that is, by a molecular‐sieve effect. (e) Both Ar and CO_2_ fit into the ultramicropores in between the carbon layers. (f) Ar is excluded from adsorption in the slit pores owing to the decreased pore width for carbonization at 800 °C. (g) Both Ar and CO_2_ are excluded from ultramicropore adsorption due to a further decrease in ultramicropore width.

All in all, from a combination of micropore and surface‐group analyses, it can be deduced that the influence of ultramicropores appears to be more significant than the effect of the nitrogen moieties. In fact, depending the carbonization temperature, it appears that slit pores with a variable width occur between the carbon layers and govern the gas adsorption, as is schematically depicted in Figure 8. The materials carbonized at 600 and 700 °C can adsorb both CO_2_ and Ar in rather large amounts between the carbon layers (Figure 8 e). However, the extremely slow adsorption of Ar indicates that the pore width is very close to the limit that is accessible to Ar atoms, which have a larger kinetic diameter than CO_2_. For carbonization temperatures of 800–875 °C the carbon interlayer spaces become too small to be penetrated by Ar, but they can still adsorb significant amounts of CO_2_, and this leads to excellent surface affinity towards CO_2_ (Figure 8 f). When the CNFs are carbonized at even higher temperatures (900–1100 °C), the coherence of the carbon layers becomes too strong, and even CO_2_ can no longer be adsorbed between the carbon layers (Figure 8 g). Moreover, the narrowness of the carbon‐layer slit pores also leads to excellent low‐pressure adsorption capability due to the overlap of pore‐wall potentials. The shrinkage of ultramicropore width is further supported by the fact that the pseudo‐irreversibility due to kinetic restrictions of CO_2_ adsorption, which is visible in CO_2_ sorption isotherms (Figure S6), increases with increasing carbonization temperature. If a stronger interaction between functional groups and CO_2_ molecules were responsible for the pseudo‐irreversibility, the relation would be inverse.

Since the kinetic diameters of many technically relevant gases are in the range of slit‐pore width and this carbon interlayer distance can be adjusted by choosing an appropriate carbonization temperature, it appears possible to tailor the material for many gas separation applications.

### IAST selectivity calculations

A common application for CO_2_ adsorbents is the separation of CO_2_ from flue gas, which contains 8–13 % CO_2_ and 71–73 % N_2_. To evaluate the performance of the CNFs with respect to this application, additional gas adsorption measurements with N_2_ were performed at 273 K. The adsorption isotherms of N_2_ and CO_2_ were simulated by using a Tóth model (Table S3), which provides an affinity constant for the interaction between adsorptive and adsorbent. The much lower affinity constant for N_2_ adsorption than for CO_2_ adsorption indicates a high selectivity towards CO_2_. Furthermore, the affinity constant for CO_2_ decreases with increasing carbonization temperature, which is a hint that the selectivity may decrease as well.

**Figure 9:**
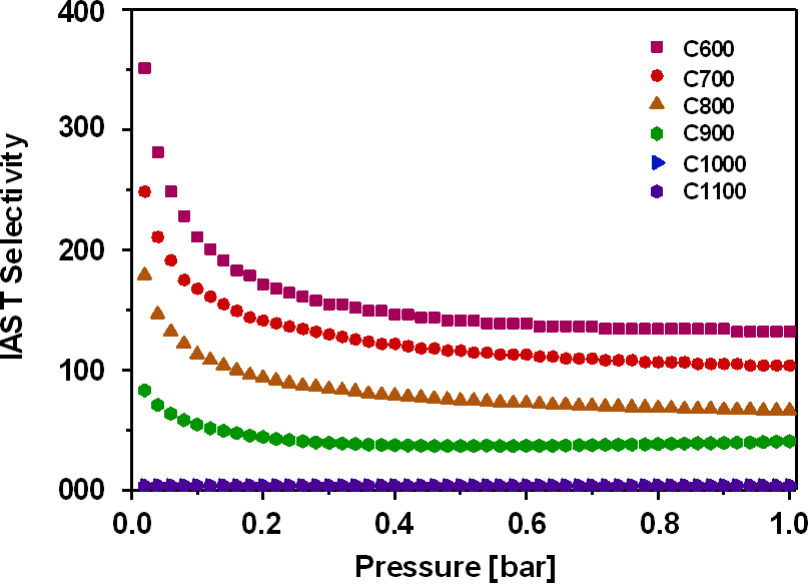
Pressure‐dependent IAST selectivity of CO_2_ over N_2_ (10:90) at 273 K for electrospun, PAN‐derived CNFs prepared at carbonization temperatures from 600 to 1100 °C (C600–C1100).

By combining the Tóth fit results of CO_2_ and N_2_ adsorption obtained at 273 K, it is possible to calculate adsorption selectivities by the IAST method. The resulting pressure‐dependent selectivities for materials carbonized between 600 and 1100 °C are plotted in Figure 9. For the material carbonized at 600 °C, the calculated IAST selectivity at 20 mbar is as high as 350. At higher pressures the selectivity drops and reaches saturation at 1 bar at a still very high value of 132, which is among the highest values reported for carbon materials in the literature so far. When carbonized at higher temperatures, the carbon fibers exhibit similar behavior of the IAST selectivity, but with lower values. For example, the IAST selectivity for a carbonization temperature of 700 °C at low pressures is as high as 250, whereas for 800 °C the material shows a selectivity of 180 at the same pressure. The plateau values at ambient pressure are high (130 and 80, respectively). In strong contrast to this, the materials carbonized at 1000 and 1100 °C exhibit IAST selectivities of less than 5.

The excellent adsorption selectivity towards CO_2_ for carbonization temperatures of 600 and 700 °C, can be explained by a molecular‐sieve effect, which is schematically depicted in Figure 8. Whereas CO_2_ can penetrate the carbon interlayer spaces, N_2_ molecules are excluded by their size, depending on the carbonization temperature of the adsorbent. In fact, the narrow ultramicropore volume, which is only accessible to CO_2_, shrinks with increasing carbonization temperature, whereas the outer fiber surface, which is accessible to both gases, does not change significantly. Hence, the selectivity towards CO_2_ decreases with increasing carbonization temperature. Furthermore, the narrowest pores with high adsorption selectivity are filled at comparatively low pressures owing to the overlap of the adsorption potentials of the pore walls, which leads to an increase in adsorption energy, which may even be enhanced by selective interaction of nitrogen functional groups and CO_2_. With increasing pressure, the rather unselective bare surface is covered, and this results in a significantly higher selectivity at lower pressures. A similar trend for the pressure dependence of the IAST selectivity has been observed by Kim et al.,^63^ whereas Zhang et al.^64^ and Wu et al.^65^ found a contrary result of drastically increasing selectivity with higher pressures. For additional comparison, the selectivities of previously reported carbons can be found in Table S3. However, none of the non‐post‐treated carbons reaches the extraordinarily high adsorption selectivity of the currently investigated carbons. The latter are only matched by tailor‐made MOFs and very few post‐treated carbons,^21^, ^66^ which potentially enables the practical application of the electrospun CNFs in gas separation processes with feed gases having relatively small CO_2_ fraction.

## Conclusions

The gas adsorption properties of PAN‐derived CNFs have been investigated. PAN fibers were prepared by electrospinning and cross‐linking at 250 °C, and carbonized at various temperatures ranging from 600 to 1100 °C. In this temperature range three different temperature regimes influencing the gas adsorption properties have been identified. From 600 to 700 °C, the resulting carbon materials can adsorb CO_2_ and Ar in large amounts. However, Ar adsorption appears to be very slow, owing to kinetic hindrance. From 800 to 875 °C, the adsorption of CO_2_ is still very high, whereas the Ar adsorption capacity decreases drastically. For carbonization temperatures of 900 °C and above, both CO_2_ and Ar adsorption become very low, owing to the significantly advancing carbonization of the investigated nanofibers. On the basis of calculated micropore size distributions, the gas adsorption properties of the CNFs are assumed to be highly dependent on the carbon interlayer distance, which is a function of the carbonization temperature and controls the access of gas molecules with different kinetic diameters, such as Ar, N_2_, and CO_2_. Owing to the narrow slit‐pore width provided by the carbon‐layer interspaces, the CNFs that were carbonized below 900 °C offer superior low‐pressure CO_2_ adsorption capabilities and extremely high IAST selectivities of up to 350. Furthermore, the influence of functional groups on the CO_2_ adsorption properties was found to be less important than that of the carbon‐layer slit pores, which act as a molecular sieve for CO_2_. A positive influence of nitrogen functional groups was found as well. The molecular‐sieve effect and the interaction between functional groups and CO_2_ make the PAN‐derived CNFs promising materials for gas adsorption and separation applications, especially for low pressures or dilute gas streams of CO_2_ such as flue gas and might be tailored even further, beyond the currently investigated gases and gas mixtures.

## Experimental Section

### Synthesis of CNFs

All chemicals were used as received without further purification. The CNFs were prepared by electrospinning of a solution containing 10 wt % PAN in DMF. In a typical synthesis, DMF (72 g, 99.8 %, VWR Chemicals, Germany) was added to PAN (8 g, *M*_w_=150 000, BOC Science, USA). The mixture was stirred at ambient temperature for 3 d until a clear solution was obtained. The solution was electrospun in an electrospinning device equipped with a rotating drum collector (IME Medical Electrospinning, The Netherlands) under constant climatic conditions of 25 °C and 30 % relative humidity in horizontal orientation. The solution was supplied by a syringe pump with a flow rate of 2.4 mL h^−1^ and pumped through a spinning needle of 0.8 mm inner diameter. The needle was moved laterally on an automated spinneret in a range of ±60 mm from the central position with a speed of 20 mm s^−1^ and a turn delay of 500 ms. The acceleration voltage was 25 kV and the needle‐to‐collector distance was 150 mm. The rotating drum collector had a diameter of 60 mm and a rotation speed of 1500 rpm. Electrospinning was performed for 6 h with a corresponding solution volume of 14.4 mL.

After electrospinning, the resulting PAN fiber mat was cut into pieces and dried in air for 1 h at 150 °C. Then, the PAN polymer chains in the fibers were cross‐linked in air at 250 °C for 15 h. Subsequently, the fibers were carbonized for 3 h in Ar atmosphere at constant temperatures ranging from 600 to 1100 °C. The heating rate of the tube furnace was 300 K h^−1^, and the cooling rate was 200 K h^−1^.

### Material characterization

XPS measurements were performed with a Phi5000 VersaProbe II (ULVAC‐Phi Inc., USA). For the individual measurements, monochromatic 1.486 keV Al_Kα_ radiation was applied. Peak analysis was performed by using CasaXPS with Shirley‐background and instrument‐specific corrections. The spectra were calibrated at the C 1s signal to 284.4 eV.

For the elemental analysis a vario EL cube elemental analyzer (Elementar, Germany) was employed. 2 mg samples of each fiber material were burned in CHN mode, and 10 mg samples in O mode. In CHN mode, the samples were burned and the combustion products were separated and detected. In O mode the samples were treated in reductive atmosphere, in which O‐containing fragments were converted to and detected as CO. This process was performed three times in both modes for each sample. For the sample of the material that was carbonized at 900 °C, polyethylene was added for better combustion.

SEM investigations were performed with a Quanta FEG 650 microscope (FEI, USA). For each image, an acceleration voltage of 20 kV was used in combination with an Everhart–Thornley detector. For the measurements, small strips of the materials were applied to the sample holder by using a copper band for additional fixation and improved electrical conductivity.

TEM images were obtained with a Titan instrument (FEI, USA). The samples were prepared by ultrasound‐mediated dispersion of the CNFs in ethanol.

Gas adsorption measurements were performed with an Autosorb iQ 2 instrument (Quantachrome, USA), which was equipped with a cryocooler (CTI‐Cryogenics, USA). The samples were prepared by cutting fiber mats into strips of 1×5 mm, 50–100 mg of which were transferred to a glass sample tube. Subsequently, the samples were degassed for 8 h under vacuum at 300 °C. For exact determination of the sample weight, the sample tubes were weighed three times in the empty state without sample. After loading with a sample the degassing process was performed and the filled tubes were weighed again three times. From the difference of the average of both masses, the sample weight was determined.

Gas adsorption measurements were performed with Ar (5.2, Air Liquide, France) at 87 K for a general pore analysis. CO_2_ (4.5, Air Liquide, France) adsorption measurements were performed at 273 K to investigate microporosity and to evaluate CO_2_ adsorption energies and selectivities. The isotherms were evaluated with regard to the surface area by the BET method.^67^ Data evaluation according to the DR equation^57^ was performed in the relative pressure range from 0.002 to 0.005 with a *β* value of 0.39. The pore size distributions were obtained by using the simulation methods provided by the measurement software Quantachrome ASiQWin 5. For Ar at 87 K a QSDFT equilibrium model (quenched solid state density functional theory, Ar on carbon, slit pores) was used, whereas for CO_2_ at 273 K an MC model (Monte Carlo, CO_2_ on carbon, slit pores) was employed. Selectivity calculations according to IAST were performed with the 3Psim software (3P instruments, Germany). Adsorption isotherms for N_2_ (5.2, Air Liquide) were measured at 273 K. The resulting isotherms as well as the CO_2_ adsorption isotherms were interpolated by using a Tóth isotherm model.^68^

## Conflict of interest

*The authors declare no conflict of interest*.

## Supporting information

As a service to our authors and readers, this journal provides supporting information supplied by the authors. Such materials are peer reviewed and may be re‐organized for online delivery, but are not copy‐edited or typeset. Technical support issues arising from supporting information (other than missing files) should be addressed to the authors.

Supplementary
