# Supplementary material for: Tailored Gas Adsorption Properties of Electrospun Carbon Nanofibers for Gas Separation and Storage
Source: ChemSusChem. 2020 May 4;13(12):3180–91. doi: 10.1002/cssc.202000520 (PMC7317881; doi:10.1002/cssc.202000520)
Supplement: Supplementary file 1 — Supplementary [file CSSC-13-3180-s001.pdf]

ChemSusChem

Supporting Information

Tailored Gas Adsorption Properties of Electrospun Carbon Nanofibers for Gas Separation and Storage

Ansgar Kretzschmar,*[a, b] Victor Selmert,[a, b] Henning Weinrich,[a] Hans Kungl,[a] Hermann Tempel,[a] and Rüdiger-A. Eichel[a, b]

Fiber morphology, structure and chemistry

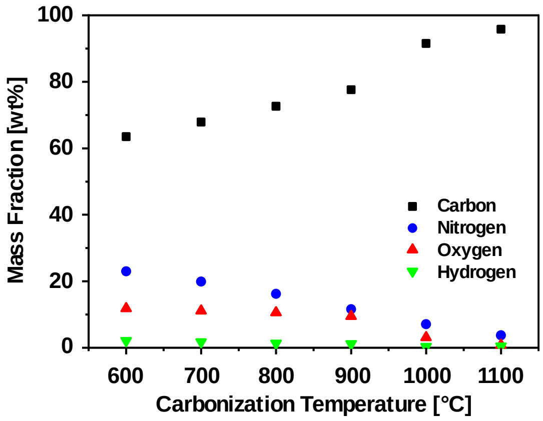

Figure S1 - Elemental composition of C600 to C1100 determined by CHNO analysis.

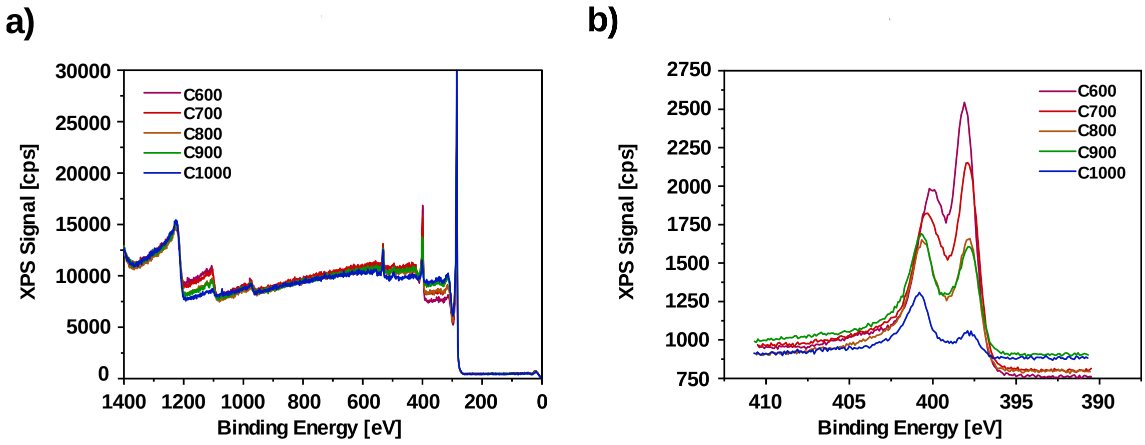

Figure S2 - Full XPS spectra (a) and N 1s peak of samples C600 - C1000 (b).

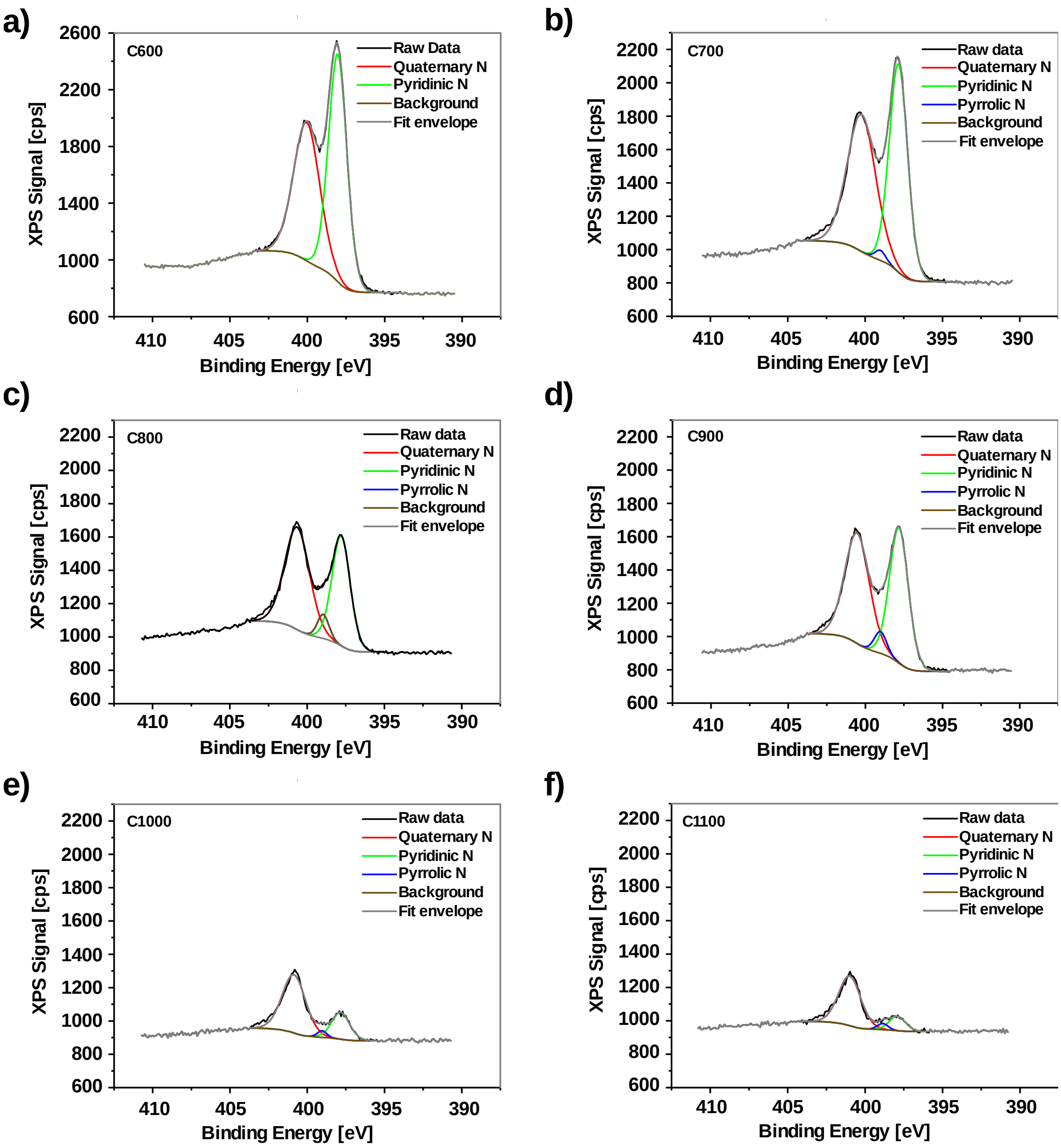

Figure S3 - XPS peak fitting for nitrogen functional groups on C600 to C1100.

Ar adsorption, surface area and pore structures

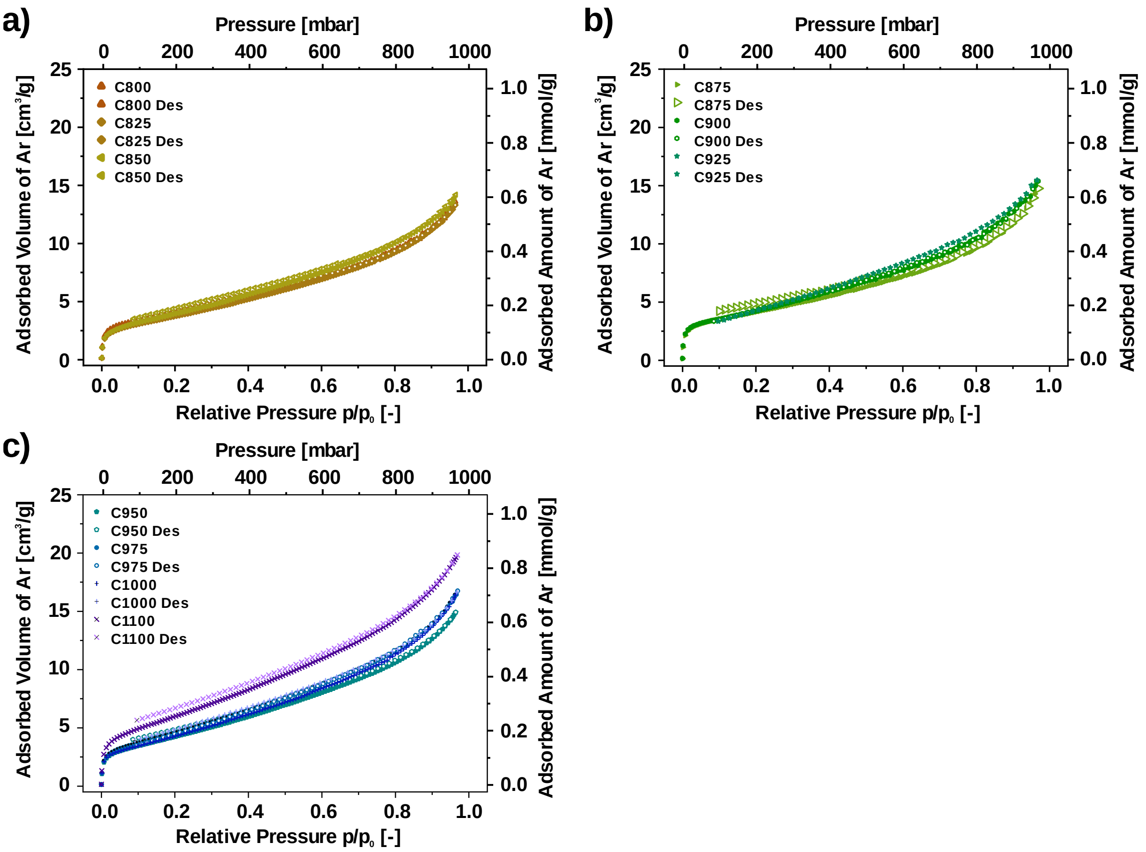

Figure S4 - Ar adsorption isotherms with desorption branches, measured at 87 K.

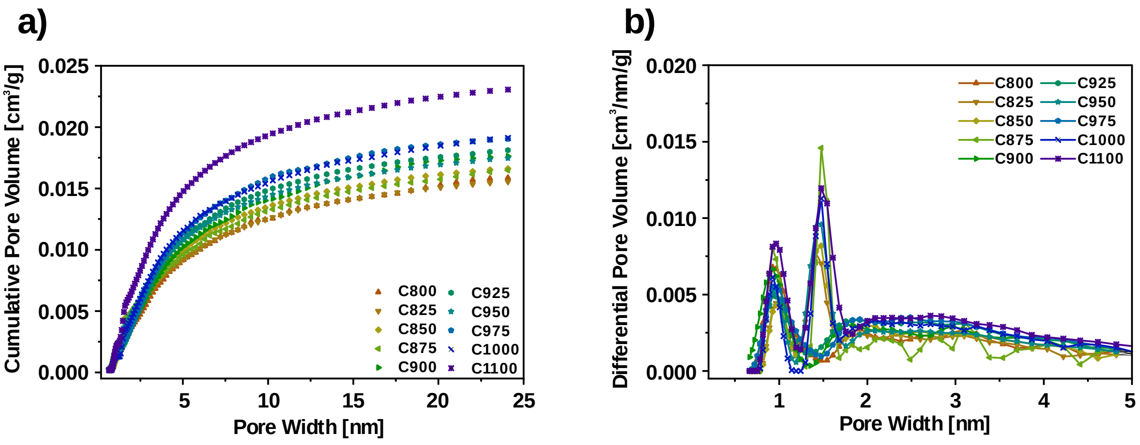

Figure S5 - Cumulative (a) and differential (b) QS-DFT pore size distributions of samples C800 to C1100 obtained from Ar-adsorption at 87 K. A reasonable calculation for C600 and C700 is not possible due to equilibration issues.

CO_2 adsorption and micropore structure

Table S1 - Comparison of the synthesized CNFs to other carbons from literature.

Adsorbed amount of CO_2 [mmol/g]				Temperature [°C]	BET area [m²/g]	$V_{ads}(CO_2)$/BET [mmol/m²]	Sample	Material	Reference
50 mbar	100 mbar	150 mbar	1000 mbar						
1.15	1.46	1.6	2.81	0 °C	259*	0.0108	C600	Carbonized electrospun PAN fibers	this work
1.17	1.49	1.7	2.82	0 °C	249*	0.0113	C700		
0.95	1.27	1.5	2.72	0 °C	13,4	0.2030	C800		
0.6	0.9	1.1	2.2	25 °C	259*	0.0085	C600		
0.7	0.9	1.1	2.2	25 °C	249*	0.0088	C700		
0.55	0.81	0.97	2.14	25 °C	13,4	0.1597	C800		
-	-	-	3.5	25 °C	1745	0.0020	K4-700		Hu et al.[1]
0.32	0.59	0.84	4.4	25 °C	2231	0.0020	PAN-PK	KOH activated PAN fibers	Shen et al.[2]
0.59	0.96	1.30	4.2	25 °C	1060	0.0040	SK-0.5-700	KOH activated soy bean dreg	Xing et al. [3]
1.71	2.32	2.68	4.5	0 °C	866	0.0052	PDA0.3/Ma0.7-2	Melamine/dopamine based carbon	Wu et al. [4]
0.78	1.21	1.47	3.64	25 °C		0.0042	PDA0.3/Ma0.7-2		
0.68	1.07	1.36	3.0	?	542	0.0055	N-AnF800(1:5)	Carbonized urea doped PAN	Kim et al. [5]
1.31	2.16	3.09	11.5	0 °C	2501	0.0046	ACM-5	Carbonized PAN monolith	Nandi et al. [6]
0.48	0.89	1.26	5.1	25 °C		0.0020	ACM-5		
0.85	1.33	1.66	4.4	0 °C	988	0.0045	CTNC-C800	Carbonized PAN/PBA copolymer	Zhong et al. [7]
0.46	0.74	0.96	3.0	25 °C		0.0030	CTNC-C800		
1.57	2.30	2.81	6.4	0 °C	1707	0.0037	CNFWs-600-2	Carbonized and activated PPy	Li et al. [8]
0.79	1.23	1.60	4.4	25 °C		0.0026	CNFWs-600-2		
1.94	2.58	3.0	6.8	0 °C	2100	0.0032	SNS2-20	KOH activated, NaOH impregnated carbon	Kim et al. [9]
1.18	1.60	1.9	4.5	25 °C		0.0021	SNS2-20		
0.45	0.73	0.97	2.76	0 °C	1134	0.0024	AC-N	Cypress Sawdust, HNO3	Zhang et al. [10]
0.22	0.38	0.53	1.73	25 °C		0.0015	AC-N		
0.94	1.62	2.16	7.32	0 °C	1825	0.0040	HPC(K-1)	Waste paper-derived carbon	Shi et al. [11]
0.47	0.86	1.23	4.68	25 °C		0.0026	HPC(K-1)		
0.76	1.27	1.65	5.41	0 °C	1730	0.0031	CNF-1	Carbon Nitride	Talapeneni et al. [12]
0.36	0.62	0.85	3.22	25 °C		0.0019	CNF-1		
0.35	0.59	0.81	2.96	5 °C	2255	0.0013	sOMC	Ordered mesoporous carbon	Yuan et al.[13]
0.20	0.36	0.49	2.00	25 °C		0.0009	sOMC		
0.83	1.25	1.54	3.31	0 °C	670	0.0049	HCM-DAH-1	Porous carbon monoliths	Hao et al.[14]
0.41	0.77	1.05	4.31	0 °C		0.0064	HCM-DAH-1-900-3		
-	1.62	-	4.04	25 °C	614	0.0066	KNC-A-K	KOH activated Nitrogen Doped carbon from DAB	Zhao et al.[15]

Values highlighted in grey have been read from isotherms.

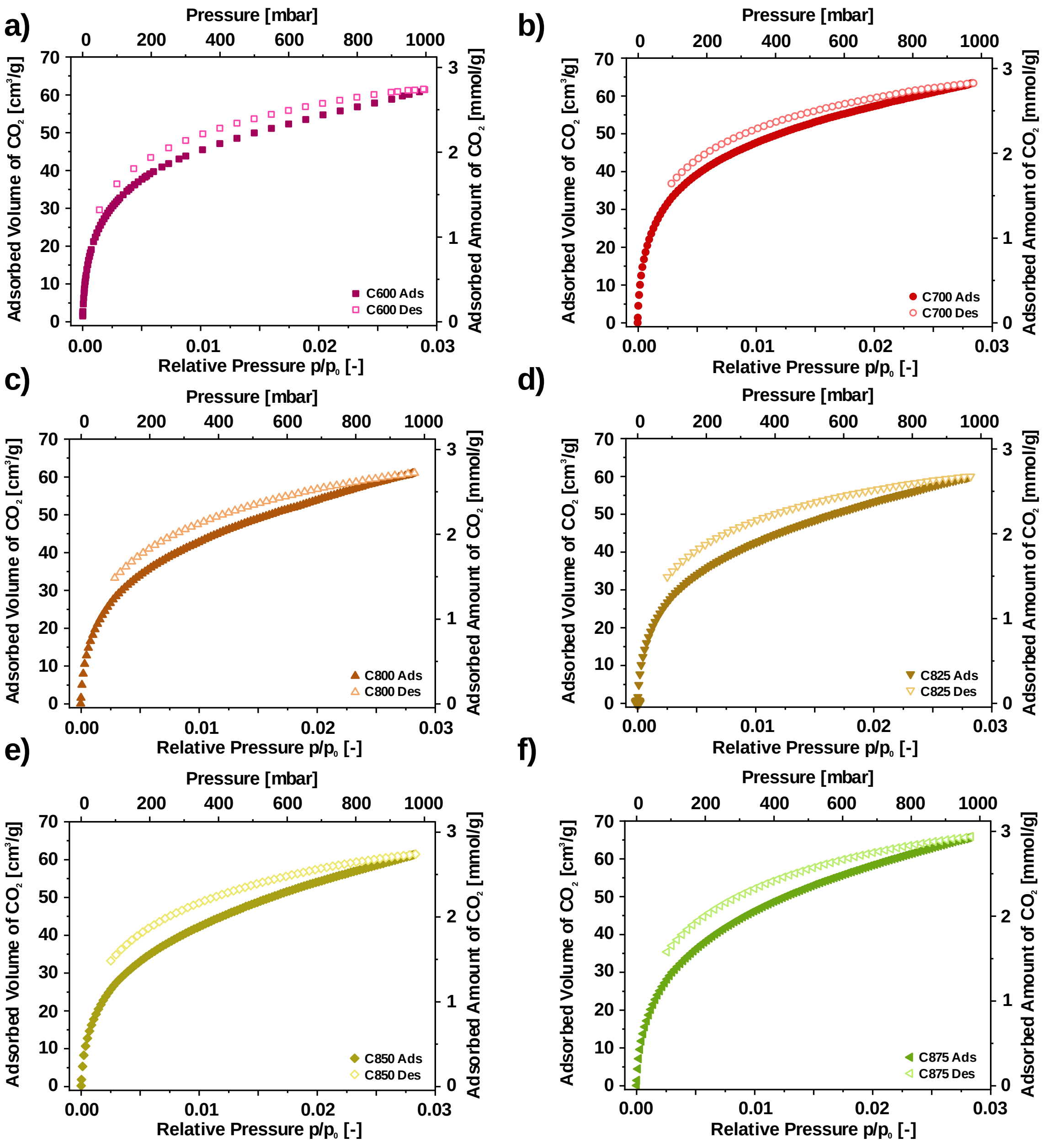

a)
b)
c)
d)
e)
f)
Pressure [mbar]
Relative Pressure p/p_0 [-]
Adsorbed Volume of CO_2 [cm³/g]
Adsorbed Amount of CO_2 [mmol/g]
C600 Ads
C600 Des
C700 Ads
C700 Des
C800 Ads
C800 Des
C825 Ads
C825 Des
C850 Ads
C850 Des
C875 Ads
C875 Des

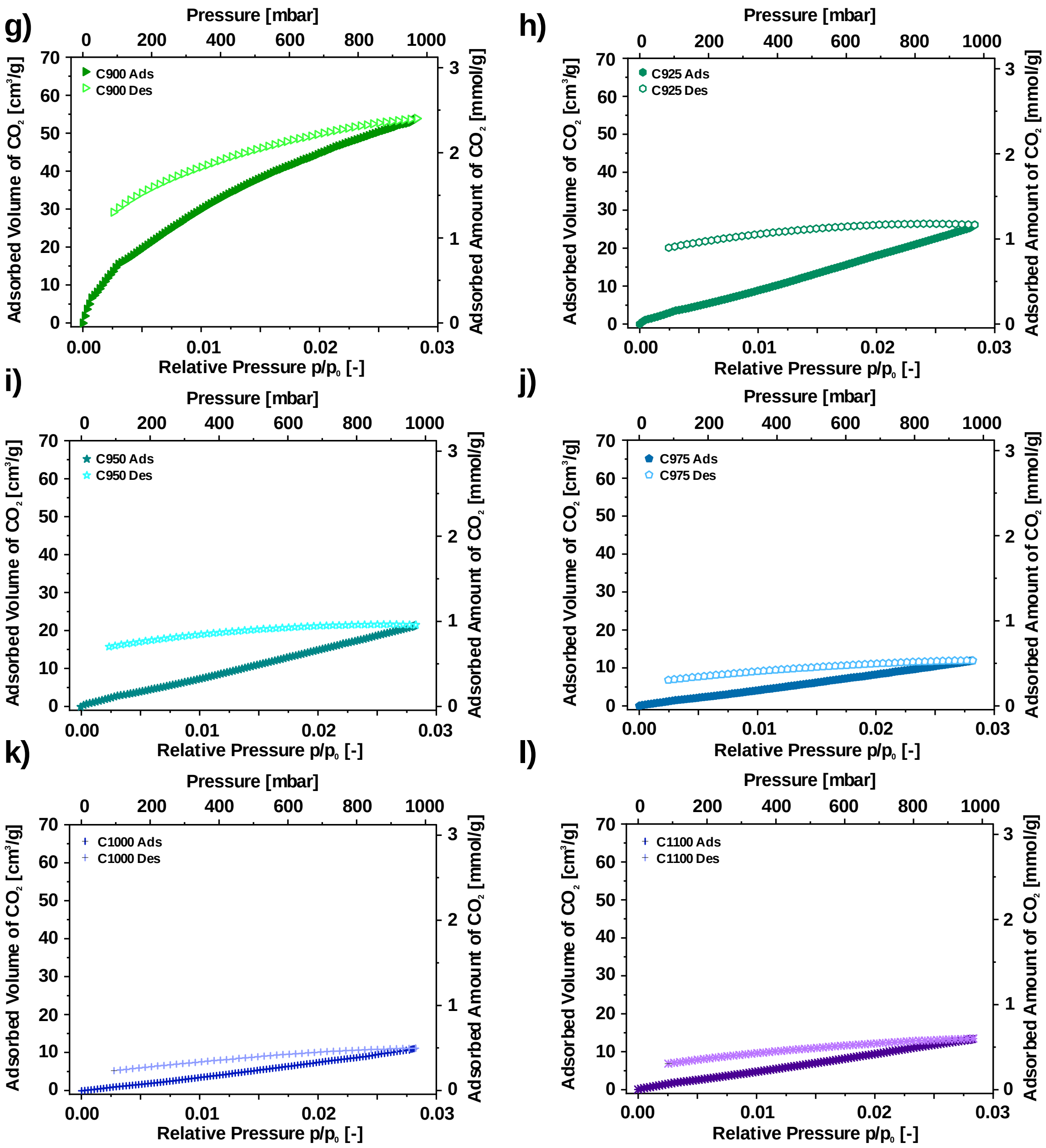

Figure S6 - CO_2 Sorption isotherms measured at 273 K with adsorption and desorption branch for all samples.

Table S2 - Adsorbed amount of CO_2 at 273 K and different pressures as well as ratio of adsorbed CO_2 vs. BET area for electrospun, PAN derived carbon nanofibers carbonized at 600 °C to 1100 °C in comparison to commercial carbon materials.

**Carboni-zation Temper-ature**	**V_{ads} (CO_2)**	**V_{ads} (CO_2)**	**V_{ads} (CO_2)**	**V_{ads} (CO_2)**	**S_{BET} (Ar)**	**V_{ads} / S_{BET}**
	50 mbar (P/P_0 = 0.00145)	*100 mbar (P/P_0 = 0.0029)*	*500 mbar (P/P_0 = 0.0145)*	*1000 mbar (P/P_0 = 0.029)*		*1000 mbar*
	[mmol/g]	[mmol/g]	[mmol/g]	[mmol/g]	[m²/g]	[mmol/m²]
600 °C	1.15	1.46	2.29	2.81	259*	0.011*
700 °C	1.17	1.49	2.35	2.82	249*	0.011*
800 °C	0.95	1.27	2.17	2.72	13.4	0.209
825 °C	0.95	1.26	2.13	2.68	12.4	0.223
850 °C	0.91	1.22	2.15	2.74	13.1	0.211
875 °C	0.96	1.34	2.34	2.93	13.2	0.226
900 °C	0.40	0.69	1.67	2.40	14.6	0.160
925 °C	0.09	0.15	0.58	1.16	15	0.078
950 °C	0.07	0.12	0.48	0.96	14.3	0.067
975 °C	0.03	0.06	0.26	0.53	16	0.033
1000 °C	0.02	0.05	0.23	0.50	14.8	0.036
1100 °C	0.04	0.07	0.30	0.60	20	0.030
SuperP®,[1]	0.03	0.08	0.45	0.99	60	0.017
Graphene Plate-lets[2] 300 m²/g	0.15	0.25	0.72	1.19	276	0.004
Black Pearls 2000®[3]	0.50	0.83	2.66	4.30	1467	0.003

*non equilibrated isotherm; [1] (Imerys); [2] (Aldrich); [3] (Cabot);

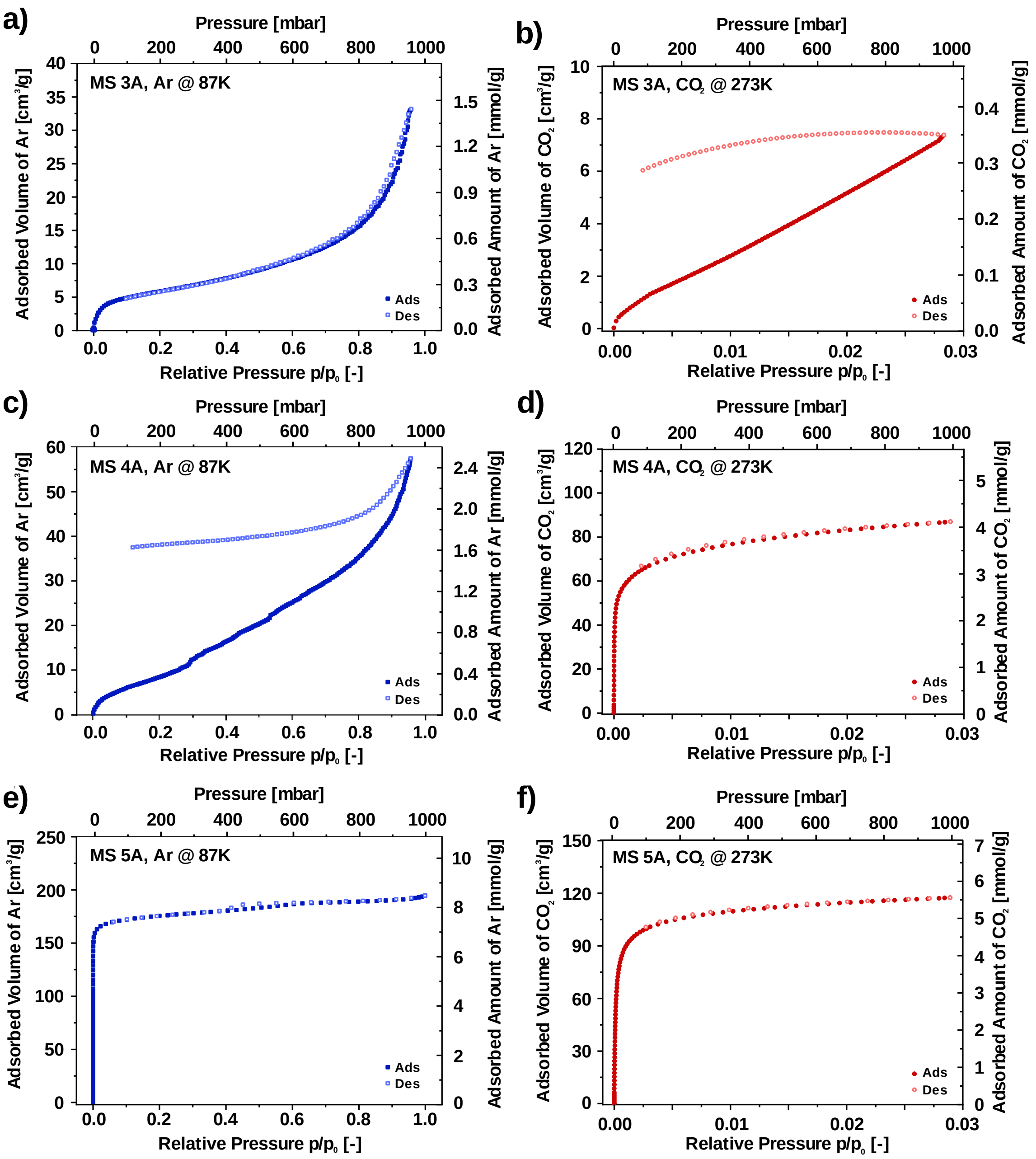

Figure S7 - Ar and CO_2 sorption isotherms of molecular sieves 3A, 4A and 5A.

The molecular sieves employed for comparison are different types of zeolite A, whose pore size can be adjusted by exchanging the cations[16].

MS3A Potassium Zeolite A

MS4A Sodium Zeolite A

MS5A Calcium Zeolite A

For MS3A, both Ar and CO_2 adsorb only in small amounts, while CO_2 exhibits pseudo irreversibility (comparable to CNFs carbonized at 1000 and 1100°C). On MS4A CO_2 can adsorb in high amount, while Ar can adsorb in medium amounts and shows pseudo irreversibility (similar to CNFs carbonized at 600-700°C). On MS5A, both CO_2 and Ar can adsorb in high amount and show reversible adsorption. The CNFs carbonized between 800 °C and 900 °C show a behavior which is a mixture of MS3A and MS4A.

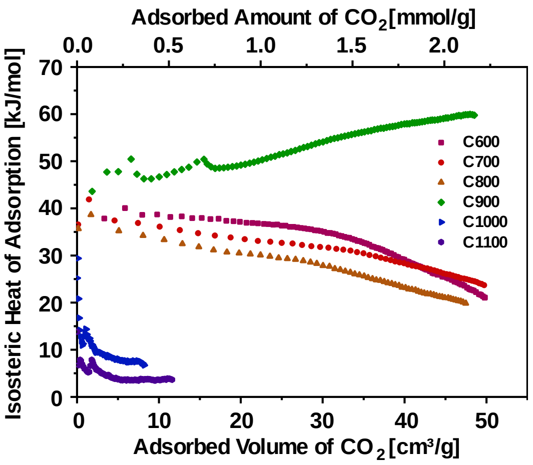

Figure S8 - isosteric heats of adsorption, calculated from CO2 adsorption isotherms measured at 273 K and 298 K. The reason for the much higher heat of adsorption for the sample carbonized at 900°C is unclear.

IAST Selectivity Calculations

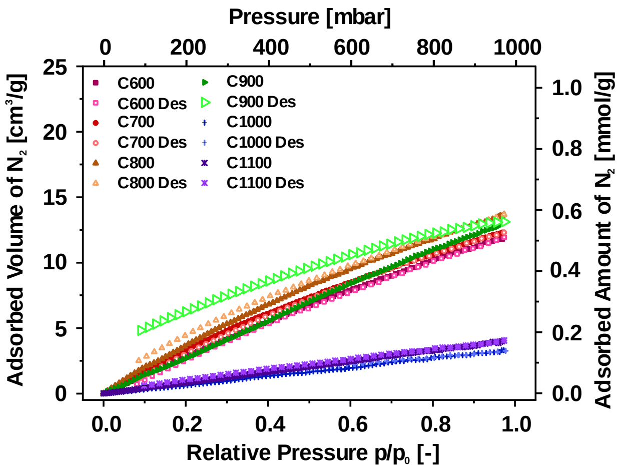

Figure S9 - N_2 sorption isotherms measured at 273K.

Tóth isotherm model

CO_2 and N_2 adsorption isotherms obtained at 273 K were modeled according to the Tóth model[17] using the Software 3P Sim (3P Instruments, Germany):

$$q_{\text{eq}} = q_{\max} \cdot \frac{K \cdot p}{\left(1 + (K \cdot p)^{t}\right)^{\frac{1}{t}}}$$

q_{eq} equilibrium loading [mmol/g]

q_{max} maximum loading [mmol/g]

K affinity constant [1/bar]

p pressure [bar]

t heterogeneity exponent [-]

Table S3 - Tóth fit parameters for the CO_2 and N_2 adsorption isotherms at 273 K for electrospun PAN derived carbon nanofibers carbonized between 600 °C and 1100 °C.

Carbonization Temperature	Measurement Gas	Affinity Constant *K* [1/bar]	Maximum loading q_{max} [mmol/g]	Heterogeneity exponent *t* [-]	R^2
600 °C	N_2 @ 273 K	0.378415	1.666549	1.4138	0.999901
700 °C		0.313893	2.617544	0.8264	0.999932
800 °C		0.429003	2.068539	0.9793	0.999926
900 °C		0.830959	0.736357	6.1214	0.999758
1000 °C		0.16534	0.894391	2.1614	0.997748
1100 °C		0.396054	0.458414	4.1047	0.999642
600 °C	CO_2 @ 273 K	204.007722	5.676074	0.273	0.999746
700 °C		118.661972	6.177126	0.286	0.999874
800 °C		109.009053	5.874381	0.2739	0.999827
900 °C		13.34087	8.921168	0.2856	0.999321
1000 °C		0.076198	6.157832	0.985	0.998087
1100 °C		0.30637	2.00154	2.2788	0.998806

Table S4 - IAST selectivities of different carbons for comparison.

IAST selectivity		Partial pressure [vol%]		Temperature [°C]	Sample	Material	Reference
100 mbar	1000 mbar	CO_2	N_2				
211	132	10	90	0	C600	N-doped CNFs from PAN	this work
64	149	10	90	25	AC-N	Cypress Sawdust, HNO3	Zhang et al.[10]
26	27	15	85	0	CNF-1	Carbon Nitride	Talapaneni et al.[12]
191	60	15	85	0	SNS2-20		
227	69	15	85	25	SNS2-20	KOH activated, NaOH impregnated carbon	Kim et al. [9]
70	38	15	85	0	HPC(K-1)		
69	41	15	85	25	HPC(K-1)	Waste paper-derived carbon	Shi et al.[11]
21	108	10	90	0	PDA0.3/Ma0.7-2		
27	115	15	85	0	PDA0.3/Ma0.7-2	Melamine/dopamine based carbon	Wu et al.[4]
10	13	50	50	5	sOMC		
9	11	50	50	25	sOMC	Ordered mesoporous carbon	Yuan et al.[13]
-	48			25	KNC-A-K	KOH activated Nitrogen Doped carbon from DAB	Zhao et al.[15]

Values highlighted in grey have been read from figures.

Further comparison

More values for CO_2 uptake at different pressures as well as values for CO_2/N_2 selectivity for comparison are given in the following references:

Oschatz et al.[18]	Table 1
To et al.[19]	Table S2
Zhao et al.[15]	Table S1
Ren et al.[20]	Table S1

[1] X. Hu, M. Radosz, K. A. Cychosz, M. Thommes, Environmental science & technology 2011, 45, 7068–7074.

[2] W. Shen, S. Zhang, Y. He, J. Li, W. Fan, *J. Mater. Chem.* **2011**, *21*, 14036.

[3] W. Xing, C. Liu, Z. Zhou, L. Zhang, J. Zhou, S. Zhuo, Z. Yan, H. Gao, G. Wang, S. Z. Qiao, *Energy Environ. Sci.* **2012**, *5*, 7323.

[4] Y. Wu, J. Wang, Y. Muhammad, S. Subhan, Y. Zhang, Y. Ling, J. Li, Z. Zhao, Z. Zhao, *Chemical Engineering Journal* **2018**, *349*, 92–100.

[5] D. W. Kim, D. W. Jung, A. A. Adelodun, Y. M. Jo, *J. Appl. Polym. Sci.* **2017**, *134*, 45534.

[6] M. Nandi, K. Okada, A. Dutta, A. Bhaumik, J. Maruyama, D. Derks, H. Uyama, *Chemical communications (Cambridge, England)* **2012**, *48*, 10283–10285.

[7] M. Zhong, S. Natesakhawat, J. P. Baltrus, D. Luebke, H. Nulwala, K. Matyjaszewski, T. Kowalewski, *Chemical communications (Cambridge, England)* **2012**, *48*, 11516–11518.

[8] Y. Li, B. Zou, C. Hu, M. Cao, *Carbon* **2016**, *99*, 79–89.

[9] Y. K. Kim, G. M. Kim, J. W. Lee, *J. Mater. Chem. A* **2015**, *3*, 10919–10927.

[10] S. Zhang, Q. Zhou, X. Jiang, L. Yao, W. Jiang, R. Xie, *Environmental technology* **2019**, 1–10.

[11] W. Shi, R. Wang, H. Liu, B. Chang, B. Yang, Z. Zhang, *RSC Adv.* **2019**, *9*, 23241–23253.

[12] S. N. Talapaneni, J. H. Lee, S. H. Je, O. Buyukcakir, T.-w. Kwon, K. Polychronopoulou, J. W. Choi, A. Coskun, *Adv. Funct. Mater.* **2017**, *27*, 1604658.

[13] B. Yuan, X. Wu, Y. Chen, J. Huang, H. Luo, S. Deng, *Environmental science & technology* **2013**, *47*, 5474–5480.

[14] G.-P. Hao, W.-c. Li, D. Qian, G.-H. Wang, W.-P. Zhang, T. Zhang, A.-Q. Wang, F. Schüth, H.-J. Bongard, A.-H. Lu, *J. Am. Chem. Soc.* **2011**, *133*, 11378–11388.

[15] Y. Zhao, X. Liu, K. X. Yao, L. Zhao, Y. Han, *Chem. Mater.* **2012**, *24*, 4725–4734.

[16] A. R. Loiola, J. C. R. A. Andrade, J. M. Sasaki, L. R. D. da Silva, *Journal of Colloid and Interface Science* **2012**, *367*, 34–39.

[17] J. Tóth, *Acta Chim. Acad. Sci. Hung.* **1962**, *35*, 416.

[18] M. Oschatz, M. Antonietti, *Energy Environ. Sci.* **2018**, *11*, 57–70.

[19] J. W. F. To et al., *Journal of the American Chemical Society* **2016**, *138*, 1001–1009.

[20] X. Ren, H. Li, J. Chen, L. Wei, A. Modak, H. Yang, Q. Yang, *Carbon* **2017**, *114*, 473–481.
